# A distributed feature selection pipeline for survival analysis using radiomics in non-small cell lung cancer patients

**DOI:** 10.1038/s41598-024-58241-1

**Published:** 2024-04-03

**Authors:** Benedetta Gottardelli, Varsha Gouthamchand, Carlotta Masciocchi, Luca Boldrini, Antonella Martino, Ciro Mazzarella, Mariangela Massaccesi, René Monshouwer, Jeroen Findhammer, Leonard Wee, Andre Dekker, Maria Antonietta Gambacorta, Andrea Damiani

**Affiliations:** 1 Department of Diagnostica per Immagini, Radioterapia Oncologica ed Ematologia, Università Cattolica del Sacro Cuore, Rome, Italy; 2 Clinical Data Science, GROW School of Oncology and Reproduction, Maastricht University, Maastricht, The Netherlands; 3 Real World Data Facility, Gemelli Generator, Fondazione Policlinico Universitario Agostino Gemelli IRCCS, Rome, Italy; 4 Department of Diagnostica per Immagini, Radioterapia Oncologica ed Ematologia, Fondazione Policlinico Universitario Agostino Gemelli IRCCS, Rome, Italy; 5 Department of Radiation Oncology, Radboud University Medical Center, Nijmegen, The Netherlands; 6 Department of Radiation Oncology (Maastro), GROW-School for Oncology and Reproduction, Maastricht University Medical Centre+, Maastricht, The Netherlands

**Keywords:** Distributed learning, Feature selection, Radiomics, NSCLC, Risk factors, Outcomes research, Data integration, Data mining, Predictive medicine, Non-small-cell lung cancer, Cancer models, Engineering

## Abstract

Predictive modelling of cancer outcomes using radiomics faces dimensionality problems and data limitations, as radiomics features often number in the hundreds, and multi-institutional data sharing is ()often unfeasible. Federated learning (FL) and feature selection (FS) techniques combined can help overcome these issues, as one provides the means of training models without exchanging sensitive data, while the other identifies the most informative features, reduces overfitting, and improves model interpretability. Our proposed FS pipeline based on FL principles targets data-driven radiomics FS in a multivariate survival study of non-small cell lung cancer patients. The pipeline was run across datasets from three institutions without patient-level data exchange. It includes two FS techniques, Correlation-based Feature Selection and LASSO regularization, and Cox Proportional-Hazard regression with Overall Survival as endpoint. Trained and validated on 828 patients overall, our pipeline yielded a radiomic signature comprising "intensity-based energy" and "mean discretised intensity". Validation resulted in a mean Harrell C-index of 0.59, showcasing fair efficacy in risk stratification. In conclusion, we suggest a distributed radiomics approach that incorporates preliminary feature selection to systematically decrease the feature set based on data-driven considerations. This aims to address dimensionality challenges beyond those associated with data constraints and interpretability concerns.

## Introduction

Creating reliable predictive models for oncology outcomes using radiomic features presents several challenges due to the lack of large amount of available data and inconclusive biological evidence of potential imaging biomarkers^1^.

Radiomics appears to be particularly suitable for the study and early diagnosis of lung cancer (LC), especially Non-Small Cell Lung Cancer (NSCLC), due to its image-rich nature and inherent heterogeneity^2^ and numerous studies have explored its potential for tailoring personalized treatments^3–10^. Radiomics constitutes a set of quantitative non-invasive biomarkers that can be calculated from medical images collected during screening, treatment, and disease monitoring phases, which are routinely gathered during ordinary clinical practice, providing a comprehensive understanding of tumour heterogeneity and predicting treatment responses^8^. By identifying distinct tumour subtypes and predicting patient-specific responses to therapies, radiomics significantly contributes to the advancement of personalized medicine in the field of lung cancer^8^. Depending on the complexity of the specific case, the number of extracted radiomic features can range from 10 to more than a thousand. This multitude of features can lead to the curse of dimensionality, making model development and interpretation difficult. Obtaining a representative sample of data becomes progressively challenging with growing dimensionality, as the available data may inadequately cover the entire feature space, introducing bias or incomplete representations of the underlying distribution and making computed statistics unreliable and predictions inaccurate. Additionally, many algorithms, based on continuous optimization (e.g., gradient descent, conjugate gradient, quasi-Newton methods), exhibit increasing time complexity as the number of dimensions increases, making computational demands impractical and yielding non optimal prediction performances^11^. Notably, in high-dimensional spaces, models contend with more parameters, increasing the risk of overfitting resulting in poor generalization to new, unseen data^12^.

To address this complexity, feature selection techniques have emerged as essential tools in the field of radiomics^13,14^. These methods help identifying the most informative and relevant features among the vast pool, reducing the risk of overfitting and enhancing the model's predictive accuracy^15^. By selecting a subset of key features, the resulting model becomes more interpretable and reliable, facilitating its clinical adoption and application^16^.

Moreover, fostering collaborations between institutions active in medical research has become of paramount importance^17,18^, but most importantly in the radiomics field, as collaborative efforts allow researchers and clinicians to pool their data resources and improve the generalizability of predictive models across different patient populations that can be scarce at a single institution level^1^. Multi-institutional studies can overcome generalizability issues due to radiomics’ sensitivity to differences in tumour delineations and image acquisition protocols and equipments^19^. In particular, in a monocentric study misleading pattern due to the data acquisition settings may contribute to the selection of non-relevant features that may not have the same predictive power in new unseen cohorts. By learning feature selection and predictive models over datasets coming from different institutions that collected data in different settings, such misleading pattern loose relevance and tend to not obfuscate the analysis and the discovery of the real clinical patterns within the whole data. In this respect, data privacy and ownership are critical concerns in collaborative research, especially when dealing with sensitive medical information^20^. Distributed, also known as federated, learning approaches play a key role to ensure privacy compliance and data ownership according to the main privacy regulations (e.g., General Data Protection Regulation—GDPR^21^) and local hospital policies^22^. In this setup, data processing and analysis can remain locally in each institution, with only aggregated results shared among collaborators. This mitigates potential privacy issues and maintains the control and data ownership by each involved party^23–26^.

Our feature selection pipeline proposes a method for the creation of robust multi-institutional models using radiomics features without necessitating the sharing of patient-level data among independent institutions, thus ensuring privacy protection throughout the process. While there have been successful studies on distributed learning applied to NSCLC^27,28^ and studies applying feature selection methods to lung cancer radiomics^14,16,29^, to the best of our knowledge, this is the first combining both approaches. We propose a distributed feature selection pipeline for building a multivariate Cox Proportional Hazard Regression model. Specifically, we developed a multivariate prediction model for overall survival (OS) using radiomics features from the original domain without prior knowledge-based selection and data from three different institutions in Europe for model training and validation. Overall Survival was selected as the primary outcome of interest, as it serves as a crucial measure in current lung cancer research and offers a reliable endpoint that can be consistently evaluated across various institutions^30^. The best radiomics feature set was defined though a data-driven feature selection approach composed with two steps: first Correlation-based feature selection^31^ and then the LASSO regularization^32^. Once the best feature set was selected through cross-validation, the final model was trained.

## Results

Overall, the conducted study included a total of 828 patients: 187 from Fondazione Policlinico Universitario “Agostino Gemelli” IRCCS (*Lung-FPG* dataset), 420 from MAASTRO Clinic (*Lung1* dataset), and 221 from Radboud UMC (*Lung2* dataset). Patients’ characteristics are presented in Table 1.

**Table 1:** Patients’ characteristics.

	Lung-FPG (n = 187)	Lung1 (n = 420)	Lung2 (n = 221)
Median age (range) at diagnosis in years	68.0 (43–99)	68.5 (34–92)	66.0 (36–87)
Median GTV size (range) in cm^3	53 (0–642)	39 (0–660)	88 (1–860)
Clinical T stage			
1–2	101 (54%)	249 (59%)	119 (54%)
3–4	83 (44%)	171 (41%)	85 (38%)
Unknown	3 (2%)	1 (0%)	17 (8%)
Clinical N stage			
0	52 (28%)	170 (40%)	49 (22%)
1	13 (7%)	22 (5%)	16 (7%)
2–3	122 (65%)	229 (55%)	137 (62%)
Unknown	0 (0%)	0 (0%)	19 (9%)
Clinical M stage			
0	177 (95%)	416 (99%)	200 (90%)
1	10 (5%)	5 (1%)	21 (10%)
Outcome			
Median follow-up in days	715	546	595
Median survival time in days	964	549	500
Death events	109 (58%)	373 (89%)	204 (93%)

We built and evaluated our proof radiomic signature using a data-driven feature selection approach using as predictors the whole set of radiomics feature extracted by PyRadiomics (v1.3) without exchanging any sensitive data via Vantage6 Federated Learning infrastructure.

Each centre was asked to reserve 20% of its cohort for global model validation, and thus a total of 166 patients was included in the validation set, while the rest 663 patients were used in the training set for model definition. Further details of the training and validation set size in each centre refer to Table S2 in Supplementary Materials.

Following the application of the Correlation-based Feature Selection (CFS) algorithm, the first step of our feature selection pipeline, five variables were automatically identified that were poorly correlated with each other and highly correlated with survival outcome. The selected variables are:

i. Mean discretised intensity from Intensity histogram features group.
ii. Root mean square intensity from Intensity-based statistical features group.
iii. 90th discretised intensity percentile from Intensity histogram features group.
iv. Intensity-based energy from Intensity-based statistical features group.
v. Flatness from Morphological features group.

**Figure 1:**
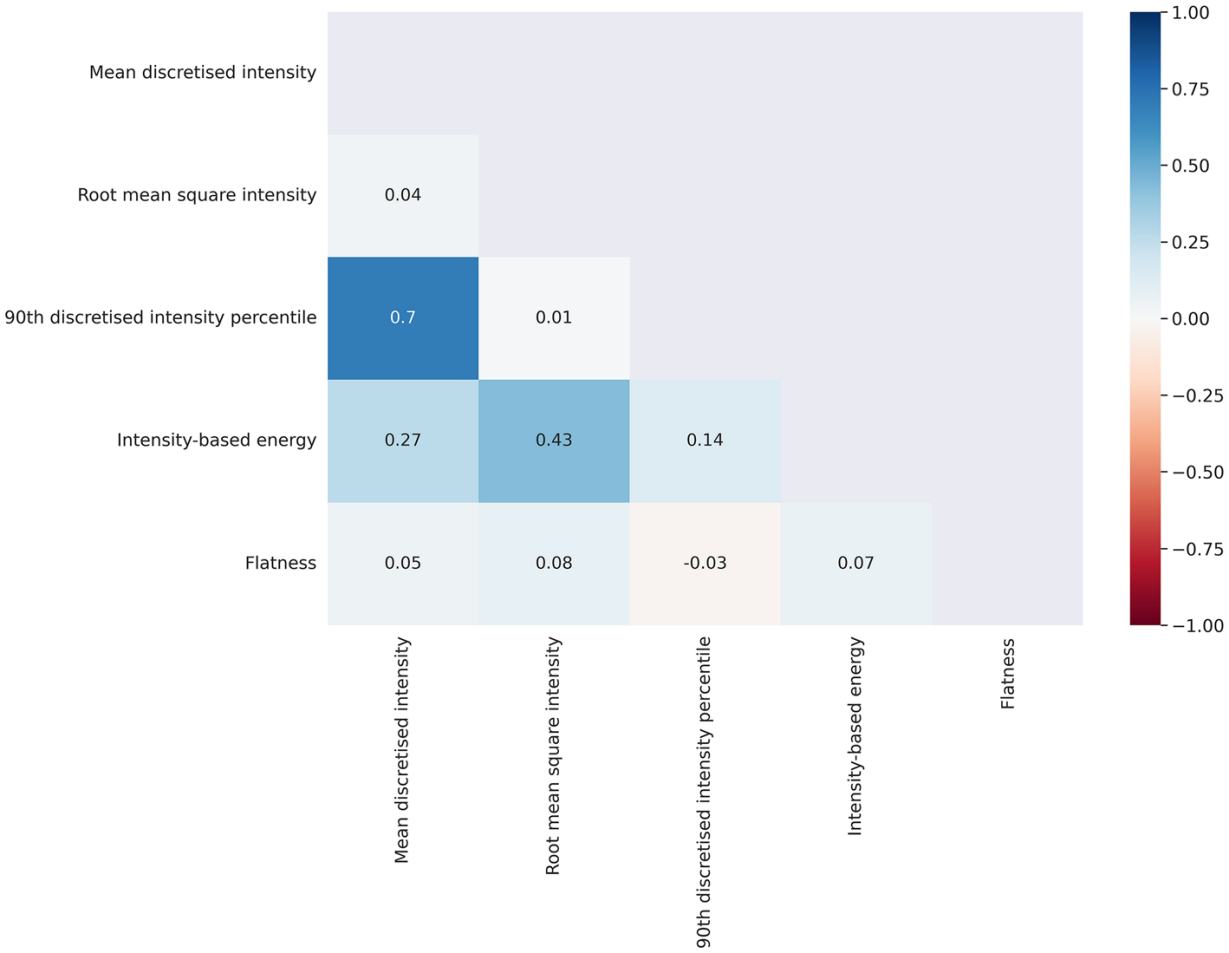
Correlation matrix of variables identified by the CFS.

Figure 1 displays the correlation matrix obtained over a distributed training. The pair of features, “mean discretised intensity” and “90th discretised intensity percentile”, exhibits the highest correlation with a Pearson's coefficient of 0.70. On the other hand, there is a slight correlation between “intensity-based energy” and "root mean square intensity”, with a Pearson's coefficient of 0.40. The remaining feature pairs show correlation coefficients close to zero. Overall features correlation with the outcome was low as the absolute median correlation coefficient was 0.05 [0.03–0.08 IQR]). All the selected features had correlation coefficients with the outcome above the 3-rd IQR, exception made for the feature “*Flatness*” (Table S3 in Supplementary Materials).

**Figure 2:**
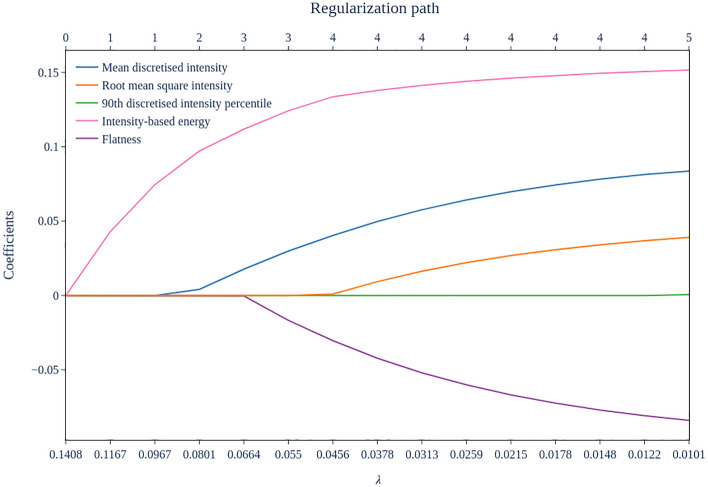
Distributed Cox regression model’s LASSO regularization path.

The distributed Cox regression model with LASSO trained, as a second step of the feature selection pipeline, with different regularisation parameter ($\lambda$) values resulted in the regularisation path shown in Fig. 2. "Intensity-based energy" results to be the most significant variable, first variable to be selected when decreasing $\uplambda$, i.e., having a non-zero coefficient. The second most important is “mean discretised intensity”, while the least important of the set is “90th discretised intensity percentile”, which is included in the model last.

The regularisation path resulted in four sets of more than one feature that were evaluated by cross validation (CV) in Leave-One-Node-Out (LONO) mode. The feature coefficients and C-index calculated at each iteration of the cross-validation steps are shown in Table 2. The best set of variables for a multivariate analysis of survival according to the cross-validation performed was that consisting of "mean discretised intensity” and "intensity-based energy", which presented an average C-index over the 3 iterations of 0.56.

**Table 2:** Feature set cross-validation.

Training nodes	Lung1	Lung-FPG	Lung2	
	Lung2	Lung1	Lung-FPG	
Validation node	Lung-FPG	Lung2	Lung1	CV C-index*
CV step: Feature set: 90th discretised intensity percentile, Mean discretised intensity, Fmorph.pca.flatness, Intensity-based energy, Root mean square intensity				
90th discretised intensity percentile	0.98 (0.83–1.15)	1.01 (0.86–1.18)	0.83 (0.67–1.04)	
Mean discretised intensity	1.08 (0.95–1.24)	1.06 (0.9–1.23)	1.91 (1.02–3.58)	
Fmorph.pca.flatness	0.93 (0.84–1.02)	0.93 (0.84–1.04)	0.88 (0.78–1.0)	
Intensity-based energy	1.17 (1.07–1.28)	1.16 (1.06–1.28)	1.24 (0.85–1.82)	
Root mean square intensity	0.94 (0.84–1.04)	1.26 (1.1–1.44)	3.25 (0.74–14.22)	
CV step c-index	0,52	0,51	0,58	0.54 [0.44–0.63]
CV step: Feature set: Mean discretised intensity, Fmorph.pca.flatness, Intensity-based energy, Root mean square intensity				
Mean discretised intensity	1.07 (0.96–1.21)	1.06 (0.95–1.2)	1.3 (0.85–1.99)	
Fmorph.pca.flatness	0.93 (0.84–1.02)	0.93 (0.84–1.04)	0.89 (0.79–1.01)	
Intensity-based energy	1.17 (1.07–1.28)	1.16 (1.06–1.27)	1.2 (0.83–1.73)	
Root mean square intensity	0.94 (0.85–1.04)	1.26 (1.1–1.44)	1.6 (0.47–5.39)	
CV step c-index	0.52	0.51	0.58	0.54 [0.44–0.63]
CV step: Feature set: Mean discretised intensity, Fmorph.pca.flatness, Intensity-based energy				
Mean discretised intensity	1.08 (0.96–1.21)	1.1 (0.97–1.23)	1.11 (0.98–1.25)	
Fmorph.pca.flatness	0.92 (0.84–1.01)	0.93 (0.83–1.03)	0.89 (0.78–1.01)	
Intensity-based energy	1.15 (1.05–1.25)	1.22 (1.12–1.33)	1.29 (0.95–1.75)	
CV step c-index	0.52	0.54	0.59	0.55 [0.46–0.64]
CV step: Feature set: Mean discretised intensity, Intensity-based energy				
Mean discretised intensity	1.07 (0.95–1.2)	1.09 (0.97–1.23)	1.11 (0.98–1.25)	
Intensity-based energy	1.14 (1.05–1.24)	1.22 (1.12–1.33)	1.31 (0.96–1.78)	
CV step c-index	0.53	0.56	0.59	0.56 [0.49–0.63]

*Mean [95% C.I.].

The results of the global Cox regression model, trained on all three nodes' training sets using as predictors “mean discretised intensity” and “intensity-based energy”, are summarised in Table 3 in the form of hazard ratio (HR) estimates. Model HRs suggest that worse overall survival is associated with higher values of both radiomic predictors.

**Table 3:** Results of the global distributed multivariate Cox regression analysis across all three centres.

	Hazard ratio (95% CI)	p-value
Intensity-based energy	1.18 (1.09–1.28)	< 0.0001
Mean discretised intensity	1.09 (1.0–1.2)	0.05

The model performance was measured on the validation set of each node individually, yielding a Harrell C-index (HCI) of 0.58 for Lung-FPG dataset, 0.61 for Lung2 dataset and 0.58 for Lung1.

**Figure 3:**
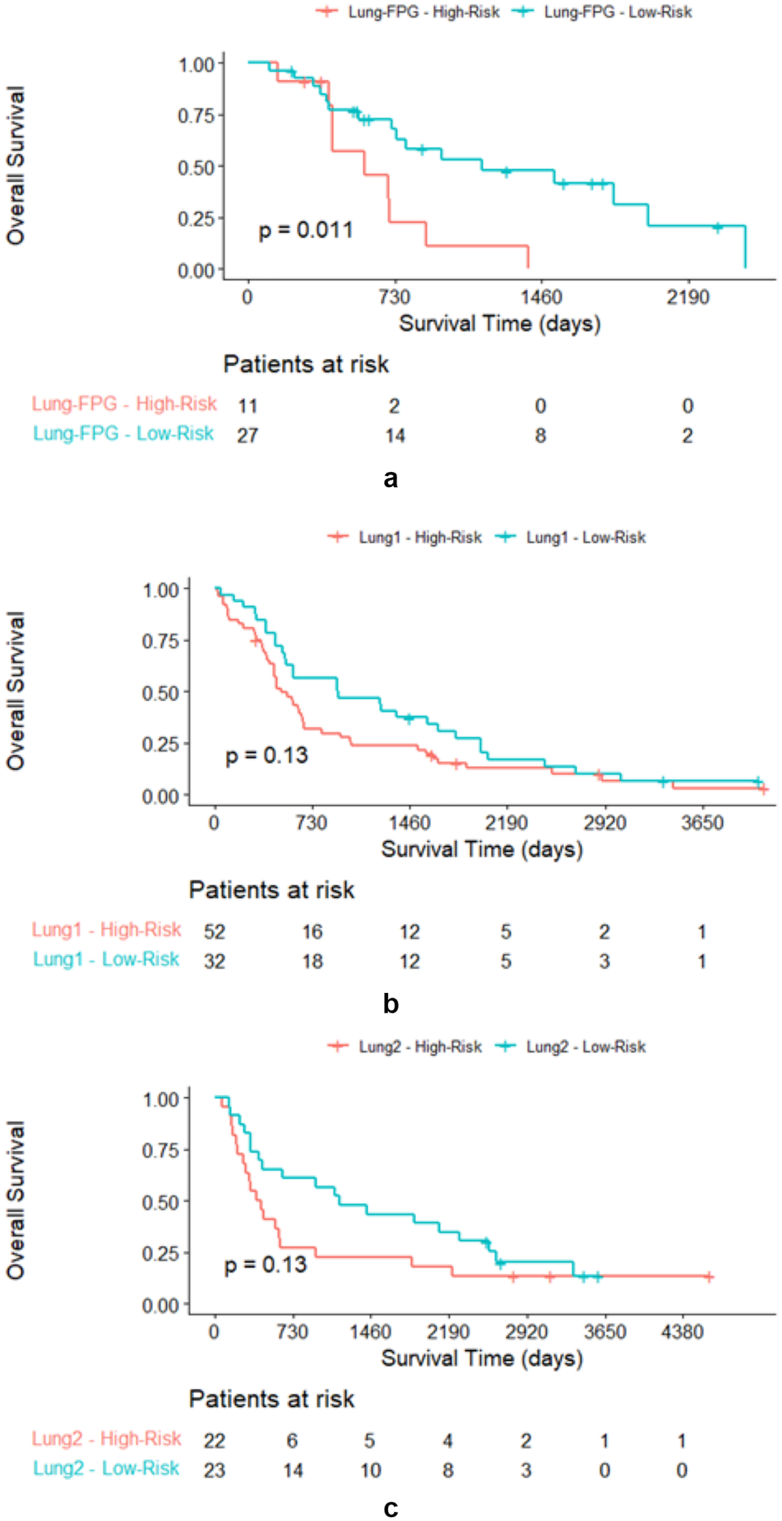
Kaplan Meier curves for high-risk and low-risk patients in Lung-FPG (**a**), Lung1 (**b**), Lung2 (**c**) validation datasets.

Using the final model, patients in the validation set (N = 167) were stratified into high-risk and low-risk classes. The estimated median risk score used as cut-off was 0.97. Patients with an individual risk score of less than 0.97 were classified as low-risk patients, while those with a higher score were assigned to the high-risk class. The low-risk group consisted of 82 patients (Lung-FPG: 27; Lung1: 32; Lung2: 23); the high-risk group consisted of 85 patients (Lung-FPG: 11; Lung1: 52; Lung2: 22). The Kaplan Meier curves in Fig. 3 show that there is a visible separation for all three datasets, being however statistically significant only the one in Lung-FPG dataset (Fig. 3a).

## Discussion

In this work we applied a distributed feature selection pipeline for the multivariate survival analysis of NSCLC patients. We trained and validated the pipeline over a distributed retrospective dataset of radiomics features from three European institutions and demonstrated robust model performance.

The novelty of our approach lies in the fact that this is the first to propose a preliminary federated feature selection phase, when compared to other published works in the field of distributed radiomics applied to lung cancer^28^. Another interesting approach for federated radiomics feature selection has been proposed by Bogowicz et al.^33^ with the aim to predict the OS of patients with head and neck cancer. In their study, feature selection is performed through intra-feature correlation calculation and the application of hierarchical clustering, while in our work we also consider the correlation of features with respect to the survival outcome using the CFS algorithm. We think that the CFS was particularly suited for the dataset due to the correlated nature of some radiomics features^34^. Moreover, we further reduce the feature set by applying LASSO regularization, that is a very well-established feature selection method^34^, to the training of Cox Regression Model to select the best feature set for the model, taking into account also the time dependent nature of the outcome. LASSO alone would have been impractical to train on a distributed network with such a large dataset, due to both the communication overhead involved in federated learning and the slow convergence of constrained optimization methods in high dimensional settings such as LASSO itself^11,35^.

LASSO made it possible to build a regularization path and to assess the feature importance over the distributed dataset. Furthermore, we consider a notable advantage of the suggested approach to be that the selection of the optimal feature set, determined through regularization, is carried out via cross-validation. This ensures a higher reliability and robustness of the observed results.

Thanks to the distributed approach we were able to train our model over 661 patients and validate it on 167, having a cohort of overall 828 patients that is well above the mean cohort size of 242.8 for this kind of studies, as reported by Ge Jie^14^. Our study could only be realised using the distributed learning methodology, which averted any need for data sharing agreements and data protection reviews.

Using the proposed methodology, we were able to reduce the set of radiomics biomarkers and identify the most important two for creating a multivariate predictive model of OS, both belonging to the first order radiomics group (intensity-based energy and mean discretised intensity). While energy results as one of the most influential features in different studies^28,36^, mean discretised intensity is more of a novelty as it is the most frequently selected feature among the first order group for classification tasks^14^.

**Figure 4:**
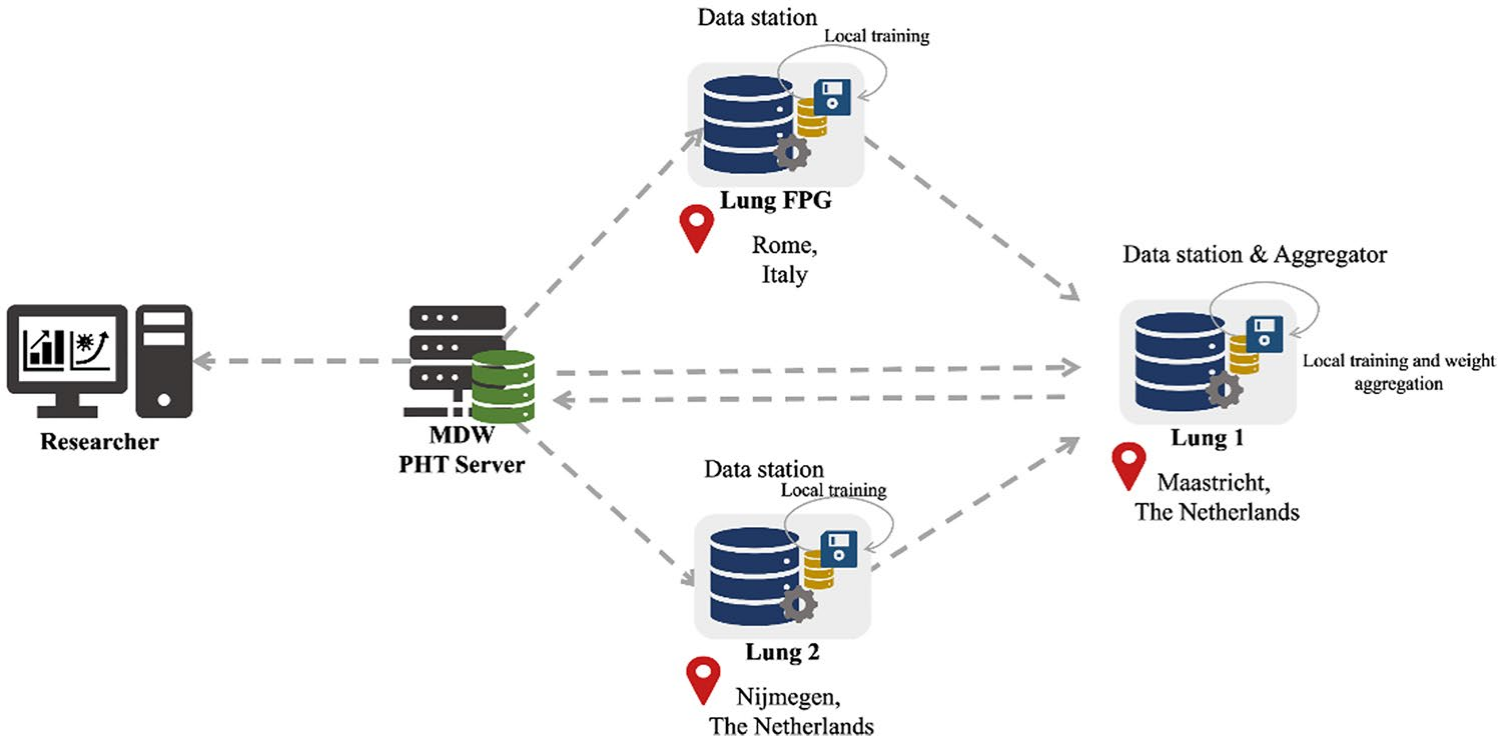
Federated learning network between Fondazione Policlinico Universitario “Agostino Gemelli” IRSS, MAASTRO Clinic and Radboud University Medical Center. (MDW: Medical Data Works).

The radiomics signature resulting from this study exhibited slightly better performance in terms of Harrel C-index than a previous study conducted by Zhenwei et al.^28^ (average HCI 0.59 vs HCI 0.58). Among the validation datasets of the three centres, the model demonstrated fair discriminative abilities between low- and high-risk patients. Even though, the Kaplan–Meiers plots in Fig. 4 show separation between the curves of the two groups in all the datasets; separation results statistically significant only for the Lung-FPG dataset (p = 0.011).

Our study was limited to the analysis of radiomics features in the original domain, motivated by the fact that it is a proof of concept of a methodology for feature selection for survival analysis rather than a study aimed at producing a definitive model to guide treatment or to test novel predictors for outcome. To develop a clinically impactful model, we believe it could be beneficial to extend the study to all domains of radiomics features. This is because some radiomics features from transformed domains (wavelet- or Laplacian of Gaussian-filtered images) have shown correlations with survival outcomes in various radiomics studies^14,37^. Moreover, it's important to note among the limitations of this study that harmonization techniques have not been employed to cover up for the typical biases of multicentric radiomics studies due to differences in the image acquisition devices or in GTV delineation^38^. We plan on expanding our distributed pipeline with such fundamental step, paving the way towards an actual clinical application of this architecture.

Furthermore, we believe that to conduct a full distributed survival analysis, the pipeline needs to be adapted to include a method to allow the calibration of the Cox regression without exchanging sensitive data^39^. At the same time, we believe it could be of great impact for building reliable survival models to study how to perform the calculation of Schoenfeld residuals on the distributed dataset to test the assumptions of Proportional Hazards for the Cox Model^40^ as we found no proposal in literature. Calculating Schoenfeld residuals and evaluating the PH assumption separately for each dataset may not reflect the characteristics of the complete dataset within which the higher feature variability may cause the assumption to fail. Finally, we want to include in the pipeline a step for building of Kaplan–Meier curves in distributed mode using Homomorphic Encryption (HE) techniques^41^. Since no preliminary aggregation is requested to each node to calculate the overall survival function, they would have to share part of their dataset, specifically survival time and outcome columns, to the aggregator node. Using HE, the aggregator node would be able to perform calculation on encrypted data without being able to retrieve the shared portions of datasets from the other nodes.

As further future work, we want to expand our distributed analysis to include both a larger network of centres and a larger set of data sources, such as genomics and clinical data other than radiomics. Having built all the pipeline for a distributed architecture based on containers such as Vantage6 makes our work reusable and deployable in any other node and prone to work with new kind of data.

## Materials and methods

The study is a retrospective multicentre distributed study conducted by the Fondazione Policlinico Universitario “Agostino Gemelli” IRCSS in Rome, Italy, in collaboration with the MAASTRO Clinic in Maastricht, the Netherlands, and the Radboud University Medical Centre in Nijmegen, the Netherlands.

Patients with pathologically confirmed primary diagnosis of NSCLC were treated with radiotherapy (3D-conformal, intensity-modulated, arc therapy, or stereotactic body radiotherapy) with or without chemotherapy. The data we employed consisted of radiotherapy planning CTs on which regions of interest were created manually by expert specialist clinicians (radiation oncologists). The segmentation of the GTV were all used without any further modification (i.e. as-is), based on the clinically approved radiotherapy plan. Pre-treatment CT scans, GTV manual delineations, clinical and survival data were available at the treating institution. The datasets from the MAASTRO Clinic and Radboud University were previously named "Lung1" and "Lung2" by Aerts et al.^42^, and the same nomenclature has been followed in this study. The dataset from the Fondazione Policlinico Universitario “Agostino Gemelli” IRCCS will be here referred to as “Lung-FPG”.

The primary focus of this study was to assess the OS as main outcome using a federated multi-institutional feature selection method for robustness. Any death, regardless of the cause, was considered an event, and patients were censored at the time of local data collection. The survival interval was determined from the date of the initial radiotherapy fraction to either the date of death or the last follow-up date if the patient was still alive.

In this proof-of-concept study, exclusively radiomics features from the original domain, extracted on unfiltered images, were employed as prognostic predictors. The extraction of these features and the image preprocessing phase were executed utilizing the PyRadiomics library (v1.3)^43^ in all the centres in order to minimise any problems of data non-homogeneity and achieve IBSI compliance^44^. PyRadiomics parameters were common to all the institutions, and they are available in the project’s public repository (https://gitlab.com/benedetta.gottardelli/total-radiomics.git). The features were derived from physician-delineated primary NSCLC tumours identified within the radiotherapy treatment planning CT images. Additionally, variables such as age and staging were extracted for descriptive analysis.

The complete list of radiomic features is provided in Table S1 in the Supplementary Materials, categorized into Morphological features, Intensity-based statistical features, Intensity histogram features, Grey level co-occurrence-based features, Grey level run length-based features, Grey level size zone-based features, Grey level distance zone-based features, and Neighbourhood grey tone difference-based features.

To ensure standardized data collection and reporting, a comprehensive ontology based on Semantic Web was shared among all participating institutions. Radiomics features and clinical data were defined by a Radiomics Ontology v1.3 (https://bioportal.bioontology.org/ontologies/RO) and a Radiation Oncology Ontology (https://bioportal.bioontology.org/ontologies/ROO), respectively, and mapped accordingly.

### Patient data collection: institutional data access and data protection approvals

Patients in Lung-FPG were treated at Fondazione Policlinico Universitario “Agostino Gemelli” IRCSS by radiotherapy for primary NSCLC between 2005 and 2018. Related data were retrieved from electronic treatment records. Tumour volumes were extracted manually from radiotherapy planning delineations. Dates of death were obtained from the electronic patient records.

Patients in Lung1 and Lung 2 dataset were treated respectively at MAASTRO Clinic and Radboud University Nijmegen Medical Centre, and related data were collected as mentioned in Aerts et al.^42^.

The investigation and findings outlined in this paper adhere to pertinent ethical guidelines and uphold proper ethical norms in the execution of research and manuscript preparation, in accordance with all relevant laws and regulations governing the treatment of human subjects. Approval for the utilization of retrospective data from individual collaborating sites has been granted by their respective institutional review boards—Comitato Etico della Fondazione Policlinico Universitario “A. Gemelli” IRCCS, MAASTRO (Dept of Radiotherapy, Maastricht University Medical Centre) Internal Review Board, Radboudumc Commissie Mensgebonden Onderzoek (CMO)—waiving specific ad-hoc patient informed consent for the study.

A consortium named “TOTAL Radiomics”, stating that no patient-level data would be shared, was formed under a signed Collaboration Agreement between Fondazione Policlinico Universitario “Agostino Gemelli” IRCCS, MAASTRO Clinic and Radboud University Nijmegen Medical Centre. In cases where necessary, local information governance and data protection reviews of the distributed learning infrastructure were obtained to ensure compliance.

### Distributed learning architecture

The distributed learning infrastructure used in this study was Vantage6 (https://distributedlearning.ai/). Vantage6 is an open-source priVAcy preserviNg federaTed leArninG infrastructurE, following the Personal Health Train (PHT) approach (https://www.health-ri.nl/initiatives/personal-health-train). Vantage6 tackles data privacy concerns by utilizing innovative privacy-preserving methods, such as bringing algorithms to the data instead of centralizing it. This approach allows organizations to collaborate on data analysis tasks without divulging raw data, sharing only aggregated cohort summaries or model coefficients. Furthermore, it enables organizations to merge datasets and insights, enhancing the development of robust and precise models^45^.

For this study, a trusted Vantage6 server was hosted at Medical Data Works (MDW), The Netherlands (Fig. 4). Three private data nodes, or “data stations”, were set up simultaneously at the three centres involved in our collaboration where the private NSCLC data were hosted. The data node hosted at MAASTRO also served as aggregator node for the models. All the three nodes were Ubuntu virtual machine instances with unique public IP addresses and distinct network firewall and were all connected to the central server. Each model built for analysis was packaged as a docker image in accordance with Vantage6 and sent as tasks to the central server using a client set up at Fondazione Policlinico Universitario “Agostino Gemelli” IRCCS in the Gemelli Generator Real Word Data facility^46^. These tasks were picked up by the three connected nodes and only the aggregated results were shared back to the client.

### Structured data conversion to semantic model

For the conversion of data from CSV (Comma Separated Values) format to a semantically rich data model, we utilized D2RQ (http://d2rq.org/). D2RQ is a mapping tool that enables the transformation of structured data into the Resource Description Framework (RDF) format, which is the cornerstone of the Semantic Web. This conversion was done by building a mapping file which specified how each table, its columns and the relationship in the data corresponds to RDF triples. Additionally, the mapping file also defines the ontologies and vocabularies for the RDF representation, allowing for semantic mappings and the establishment of meaningful relationships between entities. We then stored the resulting RDF triples in GraphDB (free version by Ontotext) running independently as a docker container in each of the node machines. GraphDB is a graph database specifically designed for managing and retrieving RDF data. Accessing data from this RDF endpoint was done using SPARQL Protocol and RDF Query Language (SPARQL).

### Distributed feature selection pipeline

The developed feature selection pipeline consists of two cascaded steps: Correlation-based Feature Selection (CFS) and LASSO regularization^32^ for Cox regression.

The CFS algorithm, originally proposed by Hall in 1999^31^, aims to identify a subset of features that exhibit strong correlation with the target variable while maintaining minimal intercorrelation among themselves. The best feature set is individuated by the CFS through a search algorithm based on a heuristic metric evaluating both the features correlation with the outcome and their intercorrelation. We adapted the CFS algorithm based on Pearsons’ regression coefficient to Vantage6 distributed learning infrastructure.

For LASSO-regularized Cox Regression model, Simon et al.^47^ proposed an optimization method based on cyclical coordinate descent which was adapted for distributed learning by Masciocchi et al.^48^. In our work, we adapted the algorithm to the Vantage6 distributed infrastructure to obtain the regularization path of the feature set derived from CFS in a distributed setting. This facilitated the establishment of a feature importance hierarchy. We compared all feature sets resulting from the regularization path, ranging from the two most important features to the inclusion of all features. The progressive inclusion of features was assessed based on the regularization path. The optimal feature set is chosen using a closed-loop “Leave-One-Node-Out" (LONO) cross-validation, where, for each feature set, new distributed Cox Regression models were trained using data from two sites and then validated on the third site in terms of Harrell’s concordance index (C-index)^49^. This was repeated three times for each feature set to cover the possible combinations.

### Model training and validation on Vantage6

**Figure 5:**
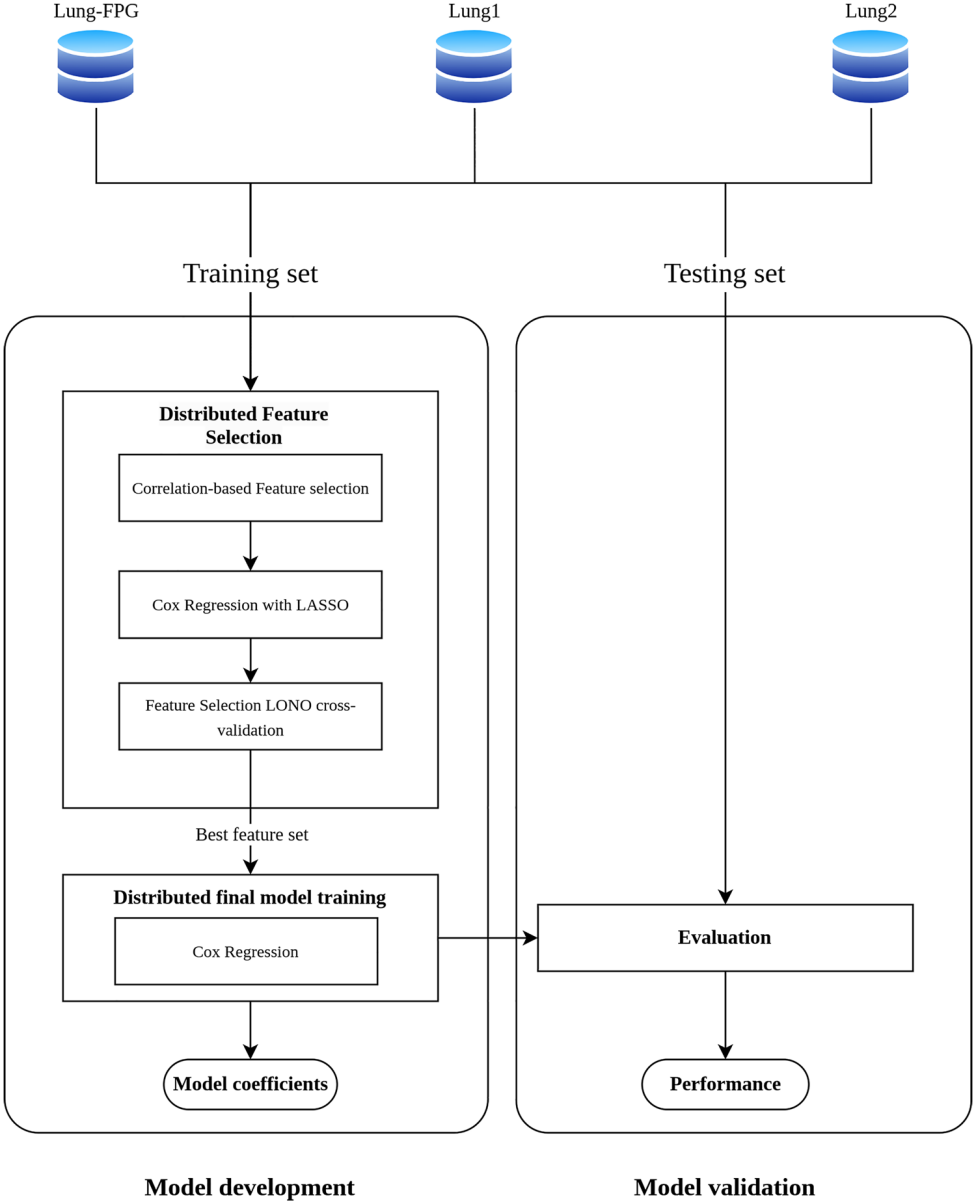
Project’s distributed analysis pipeline for feature selection on survival analysis based on radiomics feature of NSCLC patients.

The analysis steps conducted using Vantage6 distributed infrastructure are illustrated in Fig. 5. Each institution’s private data stored under RDF triples in separate GraphBDs were loaded in the Vantage6 node via SPARQL. Prior to model development, each node is instructed to randomly divide its dataset into an 80% training set and a 20% testing set for internal model validation (TRIPOD Type 2.a model development study^50^). The feature selection pipeline is then applied to the training datasets of each node. The final model's performance, measured by the C-index, was subsequently evaluated on the testing set of each node. This evaluation is performed after retraining the model on the entire distributed training dataset using the best feature set.

We conducted additional model validation to evaluate its effectiveness in stratifying risk on each centre’s testing set. To determine the individual patient risk score, we calculated the overall risk relative to the baseline by exponentiating the patient's linear predictor (LP) value. We estimated the global median risk score as the median of the medians of each centre’s risk scores from the global Cox regression model. The global median risk score was then used as a threshold to classify individual patient risk scores into high and low-risk categories. Kaplan–Meier curves were calculated at centre-level and plotted by the aggregator node.

The proposed distributed analysis pipeline was implemented using Python (v3.9.2) and adapted for the Vantage6 infrastructure (v3.7.3). The source code has been made openly accessible on GitLab at https://gitlab.com/benedetta.gottardelli/total-radiomics.git.

### Supplementary Information

Supplementary Tables.

## Data Availability

The Lung-FPG dataset supporting the findings of this work is available from the corresponding author C Masciocchi (email: carlotta.masciocchi@policlinicogemelli.it; address: Real World Data Facility, Gemelli Generator, Fondazione Policlinico Universitario Agostino Gemelli IRCCS, Largo Francesco Vito, 1, 00168 Rome, Italy) upon reasonable request in compliance with the GDPR and the Fondazione Policlinico Universitario A. Gemelli IRCCS’s policies. The images, primary tumour delineations and clinical outcomes from Lung 1 dataset supporting the findings are public open access using a Creative Commons CC-BY-NC 4.0 license, and directly available from The Cancer Imaging Archive (TCIA) under the data identifier (10.7937/K9/TCIA.2015.PF0M9REI). The Lung2 dataset that support the findings of this study are available by request from the authors R Monshouwer (email: rene.monshouwer@radboudumc.nl; address: Radboud university medical center, Department of Radiation Oncology, Geert Grooteplein 32, 6525 GA, Nijmegen, The Netherlands; phone: +31 24 361 4515). This part of data is not publicly available due to the data containing information that could compromise research participant privacy.
