# Supplementary material for: A distributed feature selection pipeline for survival analysis using radiomics in non-small cell lung cancer patients
Source: Sci Rep. 2024 Apr 3;14:7814. doi: 10.1038/s41598-024-58241-1 (PMC10991291; doi:10.1038/s41598-024-58241-1)
Supplement: Supplementary file 1 — Supplementary Tables. [file 41598_2024_58241_MOESM1_ESM.pdf]

Paper

Title

A Distributed Feature Selection Pipeline for Survival Analysis using Radiomics in Non-Small Cell Lung Cancer Patients.

Authors: Benedetta Gottardelli, Varsha Gouthamchand, Carlotta Masciocchi*, Luca Boldrini, Antonella Martino, Ciro Mazzarella, Mariangela Massaccesi, René Monshouwer, Jeroen Findhammer, Leonard Wee, Andre Dekker, Maria Antonietta Gambacorta, Andrea Damiani.

* Corresponding Author: Carlotta Masciocchi e-mail: carlotta.masciocchi@policlinicogemelli.it

Supplementary Materials

Feature Group	Feature list (n=93)
Morphological features (n=11)	Surface area (mesh), Surface to volume ratio, Sphericity, Maximum 3D diameter, Elongation, Flatness, Maximum 2D diameter (Slice), Maximum 2D diameter (Column), Maximum 2D diameter (Row), Fszm.lzhge, Fszm.glnu
Intensity-based statistical features (n=2)	Intensity-based energy, Root mean square intensity
Intensity histogram features (n=15)	Mean discretised intensity, Discretised intensity variance , Discretised intensity skewness, (Excess) discretised intensity kurtosis, Median discretised intensity, Minimum discretised intensity, 10th discretised intensity percentile, 90th discretised intensity percentile, Maximum discretised intensity, Discretised intensity interquartile range, Discretised intensity range, Intensity histogram mean absolute deviation, Intensity histogram robust mean absolute deviation, Discretised intensity entropy, Discretised intensity uniformity
Grey level co-occurrence based features (n=22)	Joint maximum, Joint average, Joint entropy, Difference average, Difference variance, Difference entropy, Sum variance, Sum entropy, Angular second moment, Contrast, Inverse difference, Normalised inverse difference, Inverse difference moment, Normalised inverse difference moment, Inverse variance, Correlation, Autocorrelation, Cluster tendency, Cluster shade, Cluster prominence, Information correlation 1, Information correlation 2
Grey level run length-based features (n=14)	Short runs emphasis, Low grey level run emphasis, High grey level run emphasis, Short run low grey level emphasis, Short run high grey level emphasis, Long run low grey level emphasis, Long run high grey level emphasis, Grey level non-uniformity, Normalised grey level non-uniformity, Run length non-uniformity, Normalised run length non-uniformity, Run percentage, Run length variance, Run entropy

Grey level size zone-based features (n=13)	Large zone emphasis, Low grey level zone emphasis, High grey level zone emphasis, Small zone low grey level emphasis, Small zone high grey level emphasis, Large zone low grey level emphasis, Normalised grey level non-uniformity, Zone size non-uniformity, Normalised zone size non-uniformity, Zone percentage, Grey level variance, Zone size variance, Zone size entropy
Grey level distance zone-based features (n=12)	Small distance emphasis, Large distance emphasis, Low grey level zone emphasis, High grey level zone emphasis, Small distance low grey level emphasis, Small distance high grey level emphasis, Large distance low grey level emphasis, Large distance high grey level emphasis, Grey level non-uniformity, Zone distance non-uniformity, Normalised zone distance non-uniformity, Grey level variance
Neighbourhood grey tone difference-based features (n=4)	Coarseness, Contrast, Busyness, Strength

Table S1: Comprehensive list of 93 radiomic features that have been utilized as predictors in our study

	N	Training set	Validation set
Lung-FPG	187	149	38
Lung1	420	336	84
Lung2	221	176	45
Total	828	661	167

Table S2: Cohort size and distribution.

Correlation coefficient	
Mean discretised intensity	0.14
Root mean square intensity	0.13
90th discretised intensity	0.13
Intensity-based energy	0.12
Flatness	-0.07

Table S3: Correlation coefficients with the outcome of variables identified by the CFS.
